# Bone Marrow Aspirate Concentrate (BMAC) for Knee Osteoarthritis: A Narrative Review of Clinical Efficacy and Future Directions

**DOI:** 10.3390/medicina61050853

**Published:** 2025-05-06

**Authors:** Dojoon Park, Hae-Seok Koh, Youn-Ho Choi, Ilkyu Park

**Affiliations:** 1 Department of Orthopaedic Surgery, St. Vincent’s Hospital, College of Medicine, The Catholic University of Korea, Suwon 16247, Republic of Korea; onedream1106@naver.com (D.P.); vincentos@naver.com (H.-S.K.); kkieek@hanmail.net (Y.-H.C.); 2 Department of Orthopaedic Surgery, Bucheon St. Mary’s Hospital, College of Medicine, The Catholic University of Korea, Bucheon 14647, Republic of Korea

**Keywords:** bone marrow aspirate concentrate (BMAC), knee osteoarthritis (OA), mesenchymal stem cells (MSCs), regenerative therapy, intra-articular injection, cartilage regeneration

## Abstract

Bone marrow aspirate concentrate (BMAC) is an autologous regenerative therapy enriched with mesenchymal stem cells (MSCs) and bioactive growth factors, offering potential disease-modifying effects in knee osteoarthritis (OA). Compared to conventional intra-articular treatments, including hyaluronic acid (HA), platelet-rich plasma (PRP), and corticosteroids, BMAC promotes cartilage regeneration, modulates inflammation, and enhances subchondral bone remodeling. Clinical evidence suggests that BMAC provides short- to mid-term symptomatic relief and functional improvement, with some studies indicating a potential to delay total knee arthroplasty (TKA). However, findings remain inconsistent, and long-term efficacy compared to PRP or autologous conditioned serum (ACS) is yet to be firmly established. Variability in BMAC preparation methods, injection protocols (single vs. repeated administration, intra-articular vs. subchondral delivery), and patient selection criteria complicates its clinical application, highlighting the need for standardized guidelines. Additionally, economic feasibility and cost-effectiveness concerns limit its widespread adoption. This review synthesizes current clinical evidence, evaluates optimal administration strategies, and explores future directions for improving treatment standardization and patient-specific therapy. Future research should prioritize well-designed, multicenter randomized controlled trials (RCTs) with long-term follow-up to confirm the sustained efficacy and therapeutic potential of BMAC in OA management.

## 1. Introduction

### 1.1. Background

Knee osteoarthritis (OA) is a major cause of disability worldwide, with its prevalence rising due to aging and obesity. This degenerative disease is characterized by cartilage degradation, subchondral bone remodeling, and chronic inflammation, leading to joint dysfunction and diminished quality of life [1,2,3]. Current treatments primarily target symptom relief rather than altering disease progression, highlighting the urgent need for regenerative therapies [4,5].

Bone marrow aspirate concentrate (BMAC) is an emerging regenerative therapy for OA. Unlike traditional intra-articular injections, such as hyaluronic acid (HA) and platelet-rich plasma (PRP), BMAC is rich in mesenchymal stem cells (MSCs) and bioactive factors that promote cartilage repair and regulate inflammation. Early studies suggest that BMAC may offer superior joint function improvement compared to some conventional regenerative treatments. However, challenges remain regarding standardization and long-term outcomes [6,7].

### 1.2. Limitations of Conventional Treatments

Current non-surgical treatments for OA, including PRP, HA, and corticosteroids, provide temporary pain relief but do not alter disease progression. However, these therapies have several limitations (Table 1).

Given these limitations, therapies that go beyond symptom relief and actively address OA pathophysiology are needed. BMAC, enriched with MSCs and bioactive factors, has been explored as a promising alternative with disease-modifying potential.

### 1.3. Overview and Advantages of Bone Marrow Aspirate Concentrate

BMAC is a bone marrow-derived formulation rich in mesenchymal stem cells (MSCs), growth factors, and a heterogeneous population of cytokines, including both pro- and anti-inflammatory mediators. Despite this diversity, BMAC exhibits regenerative and immunomodulatory properties, primarily due to the anti-inflammatory effects mediated by MSCs [11]. Unlike PRP, which primarily provides platelet-derived growth factors (PDGF), and HA, which serves as a joint lubricant, BMAC modulates multiple pathways involved in OA progression [12,13].

The preparation process involves aspiration of bone marrow—typically from the iliac crest—followed by centrifugation to concentrate cellular components, thereby increasing the viability and therapeutic activity of MSCs and bioactive factors [11,14].

Unlike platelet-rich plasma (PRP), which primarily contains platelets that release growth factors such as platelet-derived growth factor (PDGF) and transforming growth factor-beta (TGF-β), BMAC includes mesenchymal stem cells (MSCs), hematopoietic cells, and anti-inflammatory cytokines, offering broader regenerative capacity. PRP lacks the multipotent differentiation potential found in BMAC and exerts shorter-lived effects that are largely symptomatic rather than structural [1].

Hyaluronic acid (HA), meanwhile, provides mechanical lubrication and may transiently relieve pain, but it lacks the cellular and immunomodulatory properties of BMAC [15]. Autologous conditioned serum (ACS) is enriched with cytokines such as interleukin-1 receptor antagonist (IL-1Ra), but it does not contain cellular elements such as MSCs and has limited evidence of regenerative potential [1].

BMAC modulates multiple biological pathways, primarily through cartilage regeneration, inflammation suppression, and subchondral bone remodeling. These mechanisms will be further discussed in detail in the following sections.

1. Cartilage Regeneration—MSCs stimulate extracellular matrix (ECM) synthesis and promote chondrocyte differentiation [16].
2. Inflammation Modulation—Bioactive factors suppress pro-inflammatory cytokines such as IL-1β and TNF-α, mitigating joint inflammation [13].
3. Subchondral Bone Remodeling—BMAC may enhance the osteochondral interface, a critical factor in OA pathophysiology [17].

Despite its advantages, several clinical challenges persist. Variability in BMAC preparation, the absence of standardized dosing, and insufficient long-term efficacy data hinder its broader clinical application [11,14]. Current research aims to refine administration strategies and evaluate their long-term therapeutic potential.

### 1.4. Objective of This Review

This narrative review evaluates the clinical efficacy and limitations of BMAC in knee OA by addressing the following key questions:

1. Does BMAC facilitate sustained cartilage regeneration and long-term symptom relief in knee OA patients?
2. How does BMAC compare with existing intra-articular treatments in terms of clinical and economic outcomes?
3. What are the key challenges hindering BMAC’s widespread adoption, and how can these be addressed?

This review integrates evidence from clinical trials, systematic reviews, and meta-analyses to address these questions. Additionally, it explores optimal administration strategies, including single versus multiple injections, and considers economic factors affecting BMAC adoption. By consolidating current knowledge, this review offers a comprehensive evaluation of BMAC’s therapeutic potential, identifies gaps in the literature, and supports clinical decision-making in knee OA management.

### 1.5. Review Methodology

This narrative review was conducted to comprehensively analyze the current clinical applications, limitations, and future directions of BMAC in knee OA.

A systematic search of the literature was performed using PubMed, Scopus, and Web of Science, covering articles published up to February 2025. The search terms included “Bone Marrow Aspirate Concentrate AND Knee Osteoarthritis”, “BMAC AND Cartilage Regeneration”, and related keywords.

The inclusion criteria were as follows:

- Studies evaluating the clinical efficacy of BMAC in knee OA;
- Articles published in peer-reviewed journals in English;
- Randomized controlled trials (RCTs), cohort studies, systematic reviews, and meta-analyses.

The exclusion criteria were:

- Studies focusing solely on in vitro or animal models;
- Non-peer-reviewed articles, case reports, and conference abstracts.

Data were extracted and synthesized qualitatively to provide a structured review of BMAC’s current clinical applications, therapeutic potential, and limitations.

## 2. Current Clinical Applications of BMAC in Knee Treatments

### 2.1. BMAC in Knee OA

#### 2.1.1. Biological Mechanisms and Clinical Optimization of BMAC in Knee OA

BMAC has gained attention as a promising regenerative therapy for knee OA, with potential benefits in cartilage repair, inflammation modulation, and subchondral bone remodeling. These effects, driven by MSCs and bioactive factors, are central to its clinical application.

##### Cartilage Regeneration and Chondrogenesis

BMAC’s regenerative potential is linked to its MSC concentration and growth factor content, supporting chondrocyte differentiation and ECM synthesis. These effects contribute to cartilage repair and tissue integrity. Detailed mechanisms involving TGF-β, PDGF, and VEGF—shown to be elevated after BMAC administration—are further discussed in Section 2.2 [15].

##### Inflammation Modulation and Immunoregulation

BMAC modulates inflammation, a major contributor to OA progression. Although BMAC contains a variety of cytokines—including both pro- and anti-inflammatory types—its mesenchymal stem cells (MSCs) play a central role in regulating immune responses. Specifically, MSCs actively suppress key pro-inflammatory cytokines such as interleukin-1 beta (IL-1β) and tumor necrosis factor-alpha (TNF-α), thereby reducing synovial inflammation and mitigating cartilage degradation [15,18]. The precise roles of cytokines and anti-inflammatory signaling pathways are explored in Section 2.2.2

##### Subchondral Bone Remodeling

Subchondral bone integrity is crucial for joint stability and cartilage maintenance. VEGF-driven angiogenesis from BMAC may facilitate bone-cartilage interactions and structural enhancement [19]. Early studies indicate that BMAC may reduce bone marrow edema and support osteochondral repair; however, further investigation is necessary to confirm its long-term effectiveness.

##### Optimization of BMAC Preparation and Delivery

BMAC’s therapeutic efficacy is strongly influenced by its preparation method. El-Jawhari et al. [20] reported that vertical centrifugation enhances MSC viability and reduces oxidative stress, improving BMAC’s regenerative capacity. Standardizing preparation techniques is essential for optimizing clinical outcomes and ensuring reproducibility.

#### 2.1.2. Clinical Evidence Supporting BMAC in Knee Osteoarthritis

Several randomized controlled trials (RCTs) and systematic reviews have evaluated BMAC’s efficacy in knee OA, focusing on its effects on pain relief and functional improvement. While some studies have directly compared BMAC with other intra-articular treatments, such as HA and PRP, most have aimed to determine whether it offers sustained benefits beyond symptom management. Although findings indicate potential advantages over conventional therapies, variability in study design and methodological inconsistencies necessitate further research.

A randomized controlled trial by Boffa et al. [13] compared BMAC with HA over a 24-month period, demonstrating superior long-term outcomes for BMAC. Both treatments provided symptom relief; however, BMAC showed greater pain reduction, with Visual Analog Scale (VAS) scores improving more significantly at 12 months (2.2 vs. 1.7, *p* = 0.041) and 24 months (2.2 vs. 1.4, *p* = 0.002). Additionally, BMAC-treated patients exhibited sustained functional improvement, as reflected by International Knee Documentation Committee (IKDC) scores, whereas HA recipients experienced a decline over time.

A systematic review by Keeling et al. [21] evaluated 299 knees, reporting that BMAC significantly improved pain and function at short- to mid-term follow-ups. However, its efficacy was comparable to PRP and HA, underscoring the need for standardized patient selection and treatment protocols. Similarly, Di Matteo et al. [11] analyzed 1386 patients, confirming BMAC’s safety but highlighting inconsistencies in study methodologies and preparation techniques.

A placebo-controlled trial by Shapiro et al. [22] reported no significant difference in pain reduction between BMAC and saline injections (*p* > 0.09), suggesting that BMAC’s efficacy may vary across patient populations. This study was conducted in a unique within-subject design, where 25 patients received BMAC in one knee and saline in the contralateral knee, with both knees evaluated as independent controls. The study population included patients with mild to moderate OA (Kellgren-Lawrence grade I–II), and the follow-up was limited to 6 months. While the study confirmed safety, the absence of significant differences in outcomes may be attributed to the limited OA severity and relatively short observation period.

However, recent long-term studies have suggested that BMAC may still offer benefits in select patient groups. A 4-year prospective study by Pabinger et al. [23] in patients with severe knee OA (Kellgren-Lawrence grade III–IV) demonstrated substantial and sustained improvements in IKDC (from 56 ± 12 to 73 ± 13, *p* < 0.001) and Western Ontario and McMaster Universities Osteoarthritis Index (WOMAC) (from 40 ± 23 to 18 ± 18, *p* < 0.001) scores, with a 95% success rate. Notably, none of the patients required knee replacement surgery during the follow-up period, suggesting that BMAC may help delay or prevent the need for total knee arthroplasty (TKA). Unlike Shapiro’s study, this investigation did not include a control group but featured a longer follow-up and a more advanced OA population, potentially explaining the greater observed efficacy. Furthermore, the study implemented statistical controls for potential confounders such as age, BMI, and time effects. Similarly, Subramanyam et al. [24] found that 95% of patients achieved complete pain relief after one year of BMAC treatment, with significant improvements in functional outcomes (*p* < 0.0001). This prospective observational study involved a larger sample size (132 knees) and targeted patients with moderate OA (KL grade II–III). The favorable outcomes, although not compared to a placebo or alternative intervention, underscore BMAC’s clinical potential in earlier stages of disease when tissue responsiveness may be higher.

Current evidence indicates that BMAC may offer advantages over conventional treatments. However, variability in study designs and methodological inconsistencies underscore the need for standardized patient selection criteria and treatment protocols to ensure reproducible outcomes. Table 2 summarizes key findings from several studies evaluating the efficacy of BMAC in knee OA.

#### 2.1.3. BMAC for Advanced OA and Subchondral Applications

Beyond intra-articular applications, BMAC has been explored for subchondral administration, particularly in patients with advanced knee OA. Kon et al. [14] demonstrated that combined intra-articular and subchondral BMAC injections led to significant functional improvement. Magnetic Resonance Imaging (MRI) findings showed a reduction in bone marrow edema, suggesting a potential disease-modifying effect.

In patients with severe knee OA (Kellgren-Lawrence grades III–IV), Pabinger et al. [23] reported substantial and sustained clinical benefits over a 4-year follow-up period. WOMAC scores decreased from 40 to 18 (*p* < 0.001), and IKDC scores improved from 56 to 73 (*p* < 0.001). Notably, none of the patients required TKA during the study, indicating that BMAC may help delay or potentially prevent the need for surgical intervention in advanced OA cases.

These findings highlight BMAC’s emerging role in knee OA treatment, particularly in cases involving subchondral bone pathology. However, inconsistencies in study designs and patient selection underscore the need for refined stratification frameworks and longer-term data to clarify its structural impact.

### 2.2. BMAC in Cartilage Repair

#### 2.2.1. Clinical Efficacy of BMAC in Cartilage Repair

BMAC has demonstrated significant potential in cartilage repair, particularly when combined with surgical interventions such as microfracture (MFX) and osteochondral autograft transplantation (OATS). Unlike conventional regenerative approaches, BMAC not only delivers MSCs and bioactive factors but also enhances the microenvironment necessary for cartilage healing. By promoting extracellular matrix synthesis, modulating inflammation, and improving chondrogenic signaling, BMAC supports both structural regeneration and long-term joint function restoration.

##### BMAC-Enhanced Surgical Procedures

Clinical studies support the role of BMAC as an adjunctive therapy in cartilage repair procedures. Jin et al. [7] reported that BMAC combined with microfracture (MFX) significantly improved International Cartilage Repair Society (ICRS) scores compared to MFX alone (7.8 ± 3.1 vs. 6.0 ± 3.6, *p* = 0.035). Similarly, Gobbi and Whyte [25] demonstrated that BMAC combined with a hyaluronic acid-based scaffold not only led to sustained pain relief and functional improvement but also showed enhanced cartilage quality, as confirmed by second-look arthroscopy.

Furthermore, Keeling et al. [21] observed that these benefits were maintained for up to 24 months post-treatment, reinforcing BMAC’s potential in long-term cartilage repair. Collectively, these findings highlight BMAC’s value in optimizing surgical outcomes for patients with cartilage defects (Table 3).

Despite these promising outcomes, inconsistencies in clinical results remain a challenge, largely due to variations in patient selection, preparation protocols, and assessment criteria. Establishing standardized guidelines for these key factors is essential to enhance reproducibility and ensure reliable comparisons across studies.

#### 2.2.2. Mechanisms of BMAC in Cartilage Regeneration

BMAC promotes cartilage regeneration through chondrocyte differentiation, ECM synthesis, and inflammatory modulation. Within the BMAC microenvironment, MSCs differentiate into chondrocytes, enhancing cartilage structural integrity and supporting the homeostasis of the articular surface. This process is mediated by growth factor signaling and cell-cell interactions, which collectively contribute to the long-term preservation of joint function [6].

##### Growth Factor Signaling in Chondrogenesis

BMAC contains key bioactive molecules, including TGF-β, PDGF, and VEGF, which play crucial roles in cartilage regeneration. TGF-β enhances chondrocyte differentiation and upregulates collagen type II synthesis, while PDGF promotes cell proliferation and ECM deposition. VEGF stimulates angiogenesis, facilitating nutrient and oxygen delivery to the repair site, which supports long-term tissue remodeling. These factors collectively establish a pro-regenerative microenvironment, essential for sustained cartilage homeostasis and structural integrity [26,27].

##### Inflammation Modulation and Cartilage Protection

BMAC exerts immunomodulatory effects by suppressing synovial inflammation and preventing chondrocyte apoptosis and matrix degradation. MSCs inhibit key pro-inflammatory cytokines, such as IL-1β and TNF-α, while simultaneously activating anabolic signaling pathways, including IGF-1 and BMP-2, which promote cartilage matrix synthesis and repair. This dual mechanism not only reduces OA-associated inflammation but also reinforces cartilage integrity, potentially delaying disease progression and preserving joint function [28].

##### Enhancing BMAC Efficacy with Biomaterials

The combination of BMAC with biomaterial scaffolds has shown promise in enhancing cartilage repair by improving cell viability, retention, and differentiation efficiency. Xu et al. [12] demonstrated that BMAC integrated with ultrapurified alginate gel significantly enhanced cartilage regeneration in preclinical models by providing a supportive three-dimensional matrix that promotes MSC adhesion, proliferation, and chondrogenic differentiation. These biomaterial-based approaches not only prolong growth factor release but also enhance the mechanical stability of the repair site, potentially improving long-term clinical outcomes. However, further clinical validation is required to standardize scaffold composition, optimize cell-scaffold interactions, and determine the most effective delivery methods for translational applications.

Integrated with surgical techniques or biomaterials, BMAC offers a mechanistically grounded approach to cartilage repair. Advancing its clinical translation will require harmonized preparation protocols and objective tools to assess regenerative outcomes.

### 2.3. Comparison with Other Treatments

#### 2.3.1. Comparative Efficacy of BMAC vs. PRP, HA

The clinical efficacy of BMAC compared to PRP, HA in knee OA treatment has been evaluated in several major studies. While some studies report superior long-term functional improvements with BMAC [3,29], others suggest no significant difference between BMAC and PRP [21,30]. These inconsistencies may arise from variations in PRP composition (leukocyte-rich vs. leukocyte-poor), differences in HA molecular weight affecting viscoelastic properties, and variability in patient selection criteria, such as OA severity and prior treatments. Table 4 provides a comparative summary of key randomized controlled trials (RCTs), meta-analyses, and retrospective studies evaluating the clinical outcomes of BMAC, PRP, and HA, focusing on pain relief, functional improvement, and overall efficacy across different treatment groups.

Unlike PRP and HA, which primarily provide symptomatic relief, BMAC offers regenerative potential, potentially slowing OA progression and promoting structural repair [3,28]. While PRP is more accessible and minimally invasive, its effects are typically short-lived, whereas BMAC may yield sustained clinical benefits. Establishing standardized comparative trials is essential to refine patient selection criteria and develop clear efficacy benchmarks for regenerative therapies [33,34].

#### 2.3.2. Long-Term Outcomes of BMAC vs. PRP and HA

The long-term efficacy of BMAC relative to PRP and HA remains an area of ongoing investigation. Some studies indicate that BMAC provides sustained clinical benefits beyond 24 months [3,29,35], whereas others suggest no significant long-term difference between BMAC and PRP [1,36].

##### Sustainability of Clinical Benefits

El-Kadiry et al. [29] reported that BMAC-treated patients maintained significant improvements in VAS and WOMAC scores beyond 24 months, while PRP-treated patients experienced a decline after 12 months. BMAC’s prolonged effects may result from its MSC content, which supports long-term tissue repair, whereas PRP primarily relies on short-lived growth factor stimulation [3,4].

However, few studies have assessed BMAC’s efficacy beyond 3–5 years, leaving its long-term disease-modifying potential uncertain. MRI-based cartilage assessment may better distinguish symptomatic relief from true structural regeneration.

Additionally, economic evaluations should compare the cost-effectiveness of BMAC versus PRP and HA. While BMAC has a higher upfront cost, its potential to delay TKA and reduce long-term healthcare expenditures warrants further investigation [24].

#### 2.3.3. BMAC vs. Emerging Regenerative Therapies (ADSCs, SVF, Umbilical Cord MSCs)

BMAC has been compared with emerging regenerative therapies, including adipose-derived stem cells (ADSCs), stromal vascular fraction (SVF), and umbilical cord-derived MSCs (UC-MSCs). While these therapies share common regenerative mechanisms, their clinical efficacy in knee OA varies due to differences in cell composition, differentiation potential, and immunogenicity.

Jeyaraman et al. [37] reported that ADSCs provided superior short-term pain relief, while BMAC offered greater long-term benefits, particularly in cartilage repair at 24 months. The superior engraftment and differentiation potential of BMAC-derived MSCs may contribute to its prolonged regenerative effects compared to ADSCs and SVF [38].

Umbilical cord-MSCs have gained interest due to their high proliferation rates and lower immunogenicity, making them a potential alternative to BMAC. Park et al. [4] compared UC-MSCs with BMAC for cartilage repair and found that both therapies led to significant clinical improvements, though their long-term effects on cartilage regeneration remain inconclusive. Similarly, ADSCs and SVF have shown promise in osteochondral repair, suggesting that these therapies could serve as viable alternatives or adjuncts to BMAC [39].

The choice between BMAC and other regenerative therapies should consider factors such as immunogenic risks, regulatory approvals, and treatment accessibility. Large-scale randomized controlled trials are needed to determine the long-term efficacy and safety profiles of these therapies in knee OA management [40].

To further illustrate the comparative advantages and limitations, Table 5 provides a summary of BMAC, PRP, HA, and ACS, highlighting their distinct sources, benefits, and challenges.

While BMAC shows comparative promise across biologic treatments, its broader clinical adoption hinges on clarifying long-term benefits, cost-effectiveness, and treatment positioning through high-quality comparative trials and real-world validation.

### 2.4. Administration Methods

The delivery method of BMAC plays a key role in its therapeutic efficacy for knee OA. Injection frequency, route, target site, and cell concentration significantly influence clinical outcomes. Ongoing research aims to refine these factors to optimize long-term treatment strategies.

#### 2.4.1. Injection Frequency: Single vs. Repeated Administration

The optimal dosing strategy for BMAC remains unclear, with clinical studies reporting variable outcomes based on injection frequency. While a single injection alleviates symptoms, repeated administrations may enhance long-term benefits in select patients.

##### Comparative Efficacy of Single vs. Repeated Injections

Both single and repeated intra-articular BMAC injections reduce pain and improve function, though their therapeutic duration differs.

Keeling et al. [21] found that a single BMAC injection reduced VAS scores by 57.4% and improved KOOS by 75.88% over 12 months, though its effects declined in advanced OA. Conversely, Shapiro et al. [41] reported that repeated injections every 4 to 12 weeks maintained MSC levels and bioactive factors, potentially enhancing cartilage regeneration.

A systematic review by Han et al. [3] showed that repeated injections yielded better patient-reported outcomes, especially in younger patients and those with mild-to-moderate OA. However, Kyriakidis et al. [42] found that a single injection provided similar short-term benefits in Kellgren-Lawrence grade II-III OA, emphasizing the need for patient-specific dosing strategies.

##### Safety Considerations and MSC Viability

Repeated BMAC injections may sustain therapeutic benefits, but concerns regarding MSC depletion and immune response persist. Cavallo et al. [43] found that repeated intra-articular BMAC administration did not increase infection risk or systemic complications. However, multiple bone marrow aspirations may reduce MSC yield, potentially limiting long-term regenerative capacity. In addition, frequent intra-articular injections may heighten immune sensitization and inflammation, particularly in patients with chronic synovitis [13].

Further studies are needed to determine optimal injection intervals that maximize regeneration while ensuring safety [3].

#### 2.4.2. Intra-Articular vs. Subchondral Injections

The therapeutic efficacy of BMAC depends on its delivery route. Intra-articular (IA) injections introduce MSCs and bioactive factors directly into the joint, whereas subchondral injections target the osteochondral unit, a critical site in OA progression.

##### Comparative Efficacy of Intra-Articular and Subchondral Injections

A randomized controlled trial by Kon et al. [44] comparing subchondral and intra-articular (IA) BMAC injections in the same patient cohort found that subchondral administration resulted in superior outcomes, including greater pain reduction (VAS: 6.4 ± 1.1 to 2.2 ± 0.9, *p* < 0.001) and increased MRI-confirmed cartilage volume (4.2% ± 2.5%).

Similarly, Kon et al. [14] reported that subchondral BMAC injections led to more sustained functional improvements in patients with Kellgren-Lawrence grade III-IV OA compared to IA injections alone. MRI analysis revealed a reduction in bone marrow edema, suggesting that targeting the subchondral bone may enhance cartilage regeneration and joint preservation.

#### 2.4.3. Standardization and Optimization of BMAC Administration

The variability in BMAC preparation and administration techniques highlights the need for standardized protocols to improve treatment consistency and clinical outcomes.

##### Key Considerations for Standardization

1. Cell Concentration and Growth Factor Profiles
  MSC concentrations vary significantly (5 × 10^6^ to 10^7^ cells/mL), as do growth factor levels, due to differences in aspiration and centrifugation techniques [14,28]. Standardized preparation methods could improve reproducibility and therapeutic efficacy [43,45,46].

2. Injection Guidance and Delivery Method
  Image-guided BMAC delivery is essential for procedural accuracy and therapeutic effectiveness, particularly for subchondral injections. Ultrasound (US) guidance has demonstrated superior precision compared to blind injections (96% vs. 78% accuracy) [47] and is endorsed by the American Academy of Orthopaedic Surgeons (AAOS) for routine use in knee injections [3]. While MRI and fluoroscopy offer high-resolution visualization, they are less accessible and more resource-intensive [3,48,49,50]. Standardizing imaging guidance—including injection depth, anatomical landmarks, and delivery route—is critical to minimize variability, reduce complications, and improve clinical outcomes [51].

3. Optimal Injection Volume and Frequency
  Recommended injection intervals range from 4 to 12 weeks, with some studies suggesting up to six months for sustained symptom relief [35,48]. While current dosing intervals vary across studies, standardized regimens have yet to be established. This issue is further discussed in Section 3.3.

4. Patient Selection Criteria
  Identifying the most responsive patient subgroups—such as those with Kellgren-Lawrence grade II-III versus grade IV OA—may optimize treatment outcomes and cost-effectiveness [52,53].

## 3. Discussion

### 3.1. Key Findings and Comparative Effectiveness

This section discusses the comparative effectiveness of BMAC, its clinical implications, and the challenges in optimizing its therapeutic application.

BMAC has been widely investigated as a regenerative therapy for knee OA. Unlike PRP and HA, which mainly provide symptomatic relief, BMAC delivers MSCs and bioactive factors that may support long-term cartilage regeneration and inflammation modulation [6,21,43]. However, clinical outcomes vary due to differences in patient selection, preparation techniques, and follow-up durations, highlighting the need for standardized protocols to enhance treatment consistency [3,29].

#### 3.1.1. Comparison of BMAC with PRP and HA

BMAC provides longer-lasting symptom relief (≥18 months) compared to PRP (6–12 months) and HA (2–6 months), likely due to its MSC-driven anti-inflammatory and cartilage-repair mechanisms [1,11]. However, some studies report similar short-term outcomes between BMAC and PRP, particularly when leukocyte-poor PRP (LP-PRP) is used [54,55]. Variability in PRP composition and HA molecular weight further complicates direct comparisons [2,56].

These findings suggest that BMAC may be more effective in moderate OA (Kellgren-Lawrence grade II–III), whereas PRP and HA remain viable options for early-stage OA (KL I–II) [57,58]. Tailoring treatment strategies based on OA severity could help optimize outcomes across different patient subgroups [20,59].

#### 3.1.2. BMAC vs. Adipose-Derived Stem Cells (ADSCs)

While BMAC and ADSCs are both regenerative therapies, they differ in cellular composition and clinical application. ADSCs contain a higher number of MSCs, whereas BMAC is enriched with bioactive growth factors (VEGF, PDGF, and TGF-β), which enhance chondrogenesis and tissue repair [22,60]. Additionally, MSCs from BMAC demonstrate superior engraftment and retention in the joint microenvironment, potentially leading to more sustained therapeutic effects than ADSC-based therapies [61]. Given these distinctions, BMAC remains a well-established autologous option with a strong safety profile, while ADSCs and SVF require further validation through long-term clinical studies [15,62].

### 3.2. Clinical Implications and Standardization Challenges

#### 3.2.1. Need for Standardized BMAC Preparation and Administration

One of the primary challenges in BMAC therapy is the lack of standardization, which contributes to variability in clinical outcomes. Unlike PRP, which undergoes a simpler centrifugation process, BMAC contains a complex mix of MSCs and bioactive factors, making consistency more difficult to achieve [60].

To improve reproducibility and clinical efficacy, a standardized two-step centrifugation protocol is recommended:

- First spin: 1200 rpm for 10 min (red blood cell separation)
- Second spin: 2000–3000 rpm for 10–15 min (MSC concentration enhancement) [25,59,63].

Higher MSC concentrations—typically above 1000–2000 CFU/mL—have been associated with superior cartilage repair and improved functional outcomes. Therefore, strategies to maximize MSC yield during BMAC preparation are crucial to enhance therapeutic efficacy [23].

For BMAC injection protocols, current best practices recommend:

- Injection volume: 5–10 mL per session (adjusted for joint size and OA severity)
- Injection frequency: Initial treatments 4–6 weeks apart, followed by maintenance injections as needed [1,24].

Standardization challenges hinder treatment reproducibility and delay the development of universal guidelines. Implementing these strategies could enhance clinical consistency, optimize therapeutic efficacy, and improve patient outcomes in knee OA management.

#### 3.2.2. Safety and Long-Term Efficacy

Autologous BMAC has minimal immunogenicity, reducing the risk of immune rejection compared to allogeneic cell-based therapies. However, repeated bone marrow aspirations may deplete MSC reserves, potentially limiting long-term regenerative capacity [21,63]. Further long-term studies are needed to determine whether BMAC offers true disease-modifying effects or primarily functions as a symptomatic therapy [2,37].

#### 3.2.3. Economic Considerations and Cost-Effectiveness

Although BMAC has a higher initial cost per injection than PRP and HA, it may offer greater long-term cost-effectiveness by reducing the need for repeated treatments and delaying progression to TKA [21,43]. However, further economic analyses are required to assess its financial feasibility in routine clinical practice.

Studies suggest that BMAC may reduce TKA rates by 30–40% over five years, potentially leading to significant healthcare savings [3,14]. Despite this, limited insurance coverage and inconsistent reimbursement policies pose major barriers to its widespread clinical adoption [11,18,35,64]. While PRP and HA often receive partial or full reimbursement, BMAC remains largely excluded, making it less accessible to patients [24,29]. To better illustrate the cost-effectiveness and clinical feasibility of BMAC compared to PRP, HA, and ACS, Table 6 provides a comparative summary of treatment costs, injection frequency, insurance coverage, and accessibility.

### 3.3. Limitations and Future Directions

#### 3.3.1. Study Heterogeneity and Short-Term Follow-Up

Most BMAC studies report only short-term outcomes (12–24 months), limiting the ability to assess its long-term efficacy. Without extended follow-up (≥5 years) and MRI-based cartilage evaluations, it remains unclear whether BMAC provides true disease-modifying effects or simply delays symptom progression [14,71]. For instance, Kon et al. [44] noted that while BMAC may postpone or prevent TKA, the short follow-up period of current studies remains a major limitation. Addressing this gap through longitudinal trials with objective imaging assessments will be critical to establishing BMAC’s role in OA disease modification.

#### 3.3.2. Long-Term Efficacy and Research Gaps

While studies such as Keeling et al. [21] suggest that BMAC may offer long-term benefits, clinical trials extending beyond five years remain scarce.

Incorporating MRI-based cartilage assessments and histological analyses may help validate the sustained regenerative effects of BMAC. Additionally, well-structured RCTs with control groups and multi-center collaborations are essential for standardizing treatment protocols and improving the reliability of long-term data.

#### 3.3.3. Comparator Selection and Lack of Standardized Control Groups

The lack of standardized comparator groups in BMAC studies complicates the assessment of its true efficacy. While some trials compare BMAC with PRP, others evaluate BMAC in combination with PRP or HA, leading to methodological inconsistencies [11,24]. To improve study reliability, future trials should implement:

- Uniform outcome measures: Standardized clinical and imaging-based assessments;
- Consistent patient selection criteria: Stratification based on OA severity;
- Extended follow-up durations: Long-term studies to evaluate sustained efficacy.

Addressing these methodological limitations will enhance BMAC research validity and provide clearer insights into its long-term therapeutic potential [3,17].

## 4. Conclusions

BMAC is increasingly recognized as a regenerative therapy for knee OA, with potential advantages over PRP and HA. Unlike PRP and HA, which primarily offer symptomatic relief, BMAC delivers MSCs and bioactive factors that support cartilage regeneration, inflammation modulation, and subchondral bone remodeling.

Despite its potential benefits, clinical outcomes vary due to inconsistencies in preparation protocols, patient selection, and administration methods. The lack of standardized guidelines limits reproducibility, and long-term data on disease modification and safety remain insufficient. Additionally, economic feasibility remains uncertain, requiring further evaluation for broader clinical adoption.

Establishing BMAC as a standardized treatment will require convergence on preparation protocols, integration of advanced biotechnologies, and thorough evaluation of long-term efficacy and cost-effectiveness. These efforts are critical to transitioning BMAC from an investigational therapy to a clinically accepted option in OA management.

## 5. Future Directions

While BMAC therapy holds significant promise for OA management, several key challenges must be addressed to optimize its clinical application and long-term viability. Future research should focus on the following areas:

Standardization of BMAC Protocols

- Determine optimal MSC concentrations for consistent therapeutic effects.
- Develop standardized centrifugation and processing protocols to improve reproducibility.
- Optimize injection strategies for intra-articular and subchondral applications based on OA severity.

Long-Term Efficacy and Safety

- Conduct large-scale RCTs with ≥5-year follow-ups to assess sustained efficacy.
- Utilize MRI-based imaging to evaluate cartilage preservation.
- Investigate potential risks of repeated injections, including immune response and MSC depletion.

Technological Advancements

- Improve MSC retention by integrating biomaterial scaffolds, as previously discussed in Section 2.2.2.
- Explore exosome-enriched or genetically modified BMAC formulations for enhanced regenerative signaling.
- Personalize treatment using patient-specific biomarkers to improve outcomes.

Cost-Effectiveness and Clinical Accessibility

- Compare BMAC’s cost-effectiveness with PRP, HA, and surgery.
- Evaluate insurance coverage feasibility and reimbursement models.
- Identify high-benefit patient subgroups to optimize resource allocation.

Optimizing Patient Selection

- Develop biomarker-based stratification to predict treatment response.
- Assess early intervention effects in mild-to-moderate OA.
- Explore adjunct therapies (e.g., exercise, weight management) to enhance BMAC efficacy.

Future research in these areas will be critical for refining BMAC therapy, improving treatment consistency, and establishing it as a standard regenerative option for knee OA.

## Figures and Tables

**Table 1:** Comparison of Conventional Non-Surgical Treatments for Knee Osteoarthritis (OA).

Treatment	Mechanism of Action	Advantages	Limitations
Platelet-Rich Plasma (PRP) [8]	Delivers platelet-derived growth factors to promote tissue healing	Autologous; potential regenerative properties	High variability in preparation; inconsistent long-term efficacy
Hyaluronic Acid (HA) [9]	Enhances joint lubrication and reduces friction	Provides temporary symptom relief	Effectiveness varies based on OA severity
Corticosteroids [10]	Suppresses inflammation for short-term pain relief	Rapid pain relief	Potential cartilage degradation with repeated use

**Table 2:** Comparative Analysis of Bone Marrow Aspirate Concentrate (BMAC), Platelet-Rich Plasma (PRP), and Hyaluronic Acid (HA) in Knee Osteoarthritis Treatment.

Study	Comparison	Sample Size	Follow-Up Period	Pain Reduction (VAS)	Functional Improvement (IKDC/WOMAC)	Statistical Significance (*p*-Value)	Key Findings
Themistocleous et al. [17]	BMAC vs. Baseline	121	6–30 months	↓ 8.33 to 4.49	↑ OKS 20.20 to 32.29	*p* < 0.001	A single BMAC injection improved pain and function
Boffa et al. [13]	BMAC vs. HA	60	24 months	↓ 2.2 at 12 M, 2.2 at 24 M	IKDC improved for BMAC and declined for HA	*p* = 0.041 (12 M), *p* = 0.002 (24 M)	BMAC had superior long-term symptom relief in mild OA
Di Matteo et al. [11]	BMAC (Review)	1386	Various	Improved in most studies	Mixed results across studies	Variable	BMAC is safe but lacks standardization
Shapiro et al. [22]	BMAC vs. Saline	25	6 months	↓ Both knees	No significant difference vs. placebo	*p* > 0.09	BMAC is safe but has no superior effect vs. saline
Subramanyam et al. [24]	BMAC vs. Baseline	132	12 months	95% pain relief	Significant functional improvement	*p* < 0.0001	Promising short-term results
Keeling et al. [21]	BMAC (Review)	299	12.9 months	VAS/NRS improved in 5 studies	No superiority over PRP or HA	Variable	BMAC is effective but costly and not superior

↑ indicates improvement or increase; ↓ indicates reduction or decrease.

**Table 3:** Comparison of Cartilage Repair Outcomes Among Different Treatments.

Study	Treatment Comparison	Follow-Up Duration	WOMAC Improvement (%)	KOOS Improvement (%)	IKDC Score Improvement (%)	VAS Reduction (%)	ICRS Score Improvement	*p*-Value
Gobbi & Whyte (2019) [25]	BMAC + HA Scaffold vs. Other	Mean 8 years (6–10)	+65%	KOOS-Pain: +64% KOOS-Symptoms: +48% KOOS-ADL: +42% KOOS-Sports: +50% KOOS-QOL: +53%	+52%	−90%	Confirmed by second-look arthroscopy	*p* < 0.001
Keeling et al. (2021) [21]	BMAC vs. PRP vs. HA	Mean 12.9 months (6–30)	Not reported	Not reported	+45%	−75%	Not explicitly stated	No significant difference between BMAC and PRP (*p* > 0.05)
Jin et al. (2021) [7]	BMAC + MFX vs. MFX alone	24 months	Not reported	Not reported	Not reported	Not reported	BMAC + MFX: 7.8 ± 3.1 vs. MFX: 6.0 ± 3.6	*p* = 0.035

**Table 4:** Comparative Clinical Studies on Bone Marrow Aspirate Concentrate (BMAC), Platelet-Rich Plasma (PRP), and Hyaluronic Acid (HA) in Knee Osteoarthritis.

Study	Design	Comparison	N	Follow-Up	Outcome Measures	Key Findings
Dulić et al. (2021) [1]	RCT	BMAC vs. PRP vs. HA	175 (111/34/30)	1, 3, 6, 9, and 12 months	VAS, WOMAC, KOOS, and IKDC	BMAC showed significantly greater improvements than PRP and HA in all measures (*p* < 0.001). PRP > HA, but not statistically significant.
Anz et al. (2020) [30]	RCT	BMAC vs. PRP	90	1–12 months	WOMAC and IKDC	Both BMAC and PRP improved outcomes significantly (*p* < 0.001). No difference between groups at any time point.
El-Kadiry et al. (2022) [29]	Retrospective Comparative	BMAC vs. PRP	39 (26/13)	Baseline–12 months	VAS, KOOS, and WOMAC	BMAC led to significantly higher improvements than PRP (VAS ↑29%, KOOS ↑54%, WOMAC ↑52%; *p* < 0.01).
Sadabad et al. (2016) [31]	Meta-analysis	PRP vs. HA	722	Mixed (up to 2 years)	WOMAC	PRP was significantly superior to HA (SMD = –0.75; 95% CI: –1.33 to –0.18). High heterogeneity noted.
Rahman et al. (2019) [32]	RCT	PRP vs. PRP + HA	34	1 week, 1 and 3 months	VAS and IKDC	Combination therapy (PRP + HA) is significantly better than PRP alone for both pain and function (*p* = 0.001).

**Table 5:** Comparison of Cell-Based Therapies in Cartilage Repair.

Therapy	Source	Key Advantages	Limitations
BMAC (Bone Marrow Aspirate Concentrate)	Bone marrow-derived MSCs	- Autologous source, reducing immune rejection - Contains bioactive growth factors (TGF-β, PDGF, and VEGF) for cartilage repair - Potential long-term chondrogenic effects	- Variability in MSC yield and quality - Requires an invasive bone marrow aspiration procedure
PRP (Platelet-Rich Plasma)	Autologous platelets from blood	- High concentration of growth factors (PDGF, TGF-β, and IGF-1) promoting tissue healing - Less invasive and easily accessible	- Short-lived effects - Highly variable due to different preparation methods
HA (Hyaluronic Acid)	Synthetic or animal-derived hyaluronic acid	- Provides joint lubrication and reduces inflammation - Minimally invasive and widely available	- No regenerative effects - Short-term symptom relief without long-term cartilage repair
ACS (Autologous Conditioned Serum)	Blood-derived cytokine-enriched serum	- Modulates inflammatory cytokines (IL-1Ra, TGF-β) to reduce OA progression - Potential benefit in inflammatory OA cases	- Limited long-term clinical data - Requires specialized preparation

**Table 6:** Cost-Effectiveness and Clinical Feasibility of Bone Marrow Aspirate Concentrate (BMAC), Platelet-Rich Plasma (PRP), Hyaluronic Acid (HA), and Autologous Conditioned Serum (ACS).

Therapy	Cost per Injection ($) [Ref]	Number of Injections Required [Ref]	Insurance Coverage [Ref]	Long-Term Cost Savings [Ref]	Accessibility [Ref]
BMAC	$1000–$3000 [21]	1–2 injections per year [21]	Limited; varies by region [21]	Potential to delay surgery [3]	Requires bone marrow aspiration [21]
PRP	$500–$2000 [65]	3–4 injections per year [66]	Partial coverage in some regions [66]	Moderate; symptom relief for 6–12 months [67]	Readily available [66]
HA	$300–$1500 [68]	1–2 injections per year [7]	Covered in most healthcare systems [68]	Low; primarily symptomatic relief [66]	Widely available [68]
ACS	$500–$2000 [69]	Not reported	Rarely covered [21]	Uncertain; limited long-term data [70]	Requires specialized processing [21]

## Data Availability

No new data were created or analyzed in this study.
